# Hydrodynamic Microparticle Separation Mechanism Using Three-Dimensional Flow Profiles in Dual-Depth and Asymmetric Lattice-Shaped Microchannel Networks

**DOI:** 10.3390/mi10060425

**Published:** 2019-06-25

**Authors:** Takuma Yanai, Takatomo Ouchi, Masumi Yamada, Minoru Seki

**Affiliations:** Department of Applied Chemistry and Biotechnology, Graduate School of Engineering, Chiba University, 1-33 Yayoi-cho, Inage-ku, Chiba 263-8522, Japan; lyushi.15m@gmail.com (T.Y.); tktm888@gmail.com (T.O.); mseki@faculty.chiba-u.jp (M.S.)

**Keywords:** microfluidic device, particle separation, hydrodymanics, microchannel, cell sorting

## Abstract

We herein propose a new hydrodynamic mechanism of particle separation using dual-depth, lattice-patterned asymmetric microchannel networks. This mechanism utilizes three-dimensional (3D) laminar flow profiles formed at intersections of lattice channels. Large particles, primarily flowing near the bottom surface, frequently enter the shallower channels (separation channels), whereas smaller particles flowing near the microchannel ceiling primarily flow along the deeper channels (main channels). Consequently, size-based continuous particle separation was achieved in the lateral direction in the lattice area. We confirmed that the depth of the main channel was a critical factor dominating the particle separation efficiencies, and the combination of 15-μm-deep separation channels and 40-μm-deep main channels demonstrated the good separation ability for 3–10-μm particles. We prepared several types of microchannels and successfully tuned the particle separation size. Furthermore, the input position of the particle suspension was controlled by adjusting the input flow rates and/or using a Y-shaped inlet connector that resulted in a significant improvement in the separation precision. The presented concept is a good example of a new type of microfluidic particle separation mechanism using 3D flows and may potentially be applicable to the sorting of various types of micrometer-sized objects, including living cells and synthetic microparticles.

## 1. Introduction

The need to separate micrometer-sized particles precisely, especially mammalian cells of specific phenotypes, is increasing with the recent progress in cell-based liquid biopsy technologies and stem cell engineering [1,2,3]. In the industrial production of synthetic microparticles, monodispersity at a particle size is a critical factor dominating the function and reliability of particle-based products, as represented by particle-based separation matrices. In the last decade, microfluidic systems have been recognized as a practical tool for precisely separating micrometer-sized cells and particles [4,5,6]. Several types of microfluidic cell separators are commercially available, most of which employ laminar flow systems or inertial forces of particle movement in microchannels. Representative examples of particle sorting mechanisms that do not necessitate the application of outer forces include deterministic lateral displacement (DLD) [7,8,9], pinched-flow fractionation (PFF) [10,11], hydrodynamic filtration [12,13,14], Dean-flow fractionation [15,16,17], hydrophoresis [18,19,20], inertial microfluidics [21,22], and multi-orifice fractionation [23,24]. Most of these techniques utilize precisely controlled flow profiles in a quasi-two-dimensional microchannel, i.e., laminar flow patterns in microchannels with a uniform depth, neglecting the flow rate distribution in the *z* (depth) direction. Meanwhile, recent studies have demonstrated that three dimensionally fabricated microchannels effectively function as new particle sorting/focusing devices [25,26]. Researchers have used secondary flows, Dean-flows, or microvortices formed in the microchannel cross section (*x*-*z* plane) [27,28,29,30]. From these examples, we expect that unprecedented but efficient microfluidic mechanisms for particle separation can be developed using three-dimensional (3D) flow profiles in microfluidic channels with non-uniform depths. 

In our recent study, we proposed the concept of a purely hydrodynamic particle separation scheme using slanted, asymmetrically arranged, lattice-shaped microchannel networks [31]. The lattice structure was composed two types of perpendicularly crossing microchannels (“main channels” and “separation channels”) with a uniform depth. To split a small amount of fluid flow from the main channel to the separation channel at each crossing point, the density of the separation channels was 30–100 times higher than that of the main channels. Using this microchannel configuration, large particles flow along the main channel, whereas small particles enter the separation channels, resulting in the size-dependent separation of particles in the lateral (*x*) direction. The lattice configuration of the microchannel was advantageous because it is robust against microchannel clogging. Additionally, the separation throughput can potentially be increased compared with single microchannels with a depth/width of several tens of micrometers. However, one concern remains: the number of main channels is limited, and only ~10 main channels can be placed in the lattice region with a width of ~10 mm. We pondered the outcome of placing multiple main channels more densely, instead of creating a significant difference in the densities of the two types of perpendicularly crossing channels. This was our primary motivation to test a new type of lattice microchannel-based particle separation technique. 

**Figure 1:**
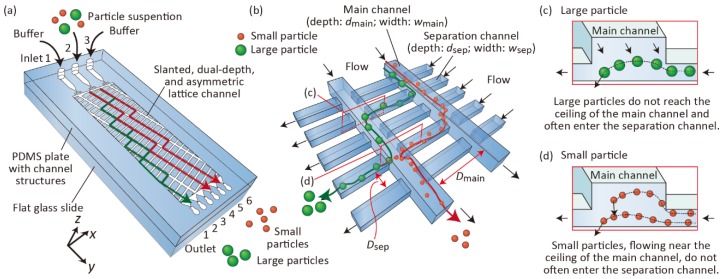
(**a**) Schematic image illustrating particle separation behaviors using dual depth, lattice-shaped channel networks. (**b**) Detailed behaviors of particles in the lattice region. (**c**,**d**) Illustrations showing the particle movement in the depth (*z*) direction from the shallower separation channels into the main channels. These images correspond to the cross sections shown in panel (**b**).

Herein, we describe a new principle of size-based particle separation using asymmetric lattice channel networks composed of two channel types that are more densely arranged and have different depths. The schematic images exhibiting the particle separation behaviors are shown in Figure 1. To create an anisotropic flow distribution at each crossing point, we fabricated dual-depth lattices; the main channels, which were slanted to the lower right direction (+ *x* and + *y* directions), were made deeper, whereas the separation channels, which were perpendicularly crossing the main channels, were shallower. From the precise observation of the particle behaviors in the channels, we noticed that complex 3D flows critically dominated the size-dependent difference in the particle behaviors, thereby achieving particle separation in the lateral direction. Particularly, it was interesting that the large particles frequently entered the shallower separation channels, whereas the small ones did not. These separation behaviors were completely different from those observed in our previous study using uniform-depth lattice channels [31], or the previously well-developed DLD techniques. In this study, we investigated several factors affecting the separation performances of particles and conducted several experiments to tune the separation size and improve the separation efficiencies. 

## 2. Materials and Methods

### 2.1. Separation Mechanism

The detailed separation mechanism is shown in Figure 1. We employed asymmetric lattice-channel networks with three inlets and six outlets. The lattice structure was composed of deep main channels and shallow separation channels (Figure 1b). We introduced a particle suspension from Inlet 2 and buffer solution from Inlets 1 and 3. Because the main channels are slightly slanted against the flow direction (*y* direction), and a small amount of the fluid flow is split from the main channel into the separation channel at every crossing point, particles alternately flow through the main channel and separation channels. At the moment when particles enter the main channel from the separation channel, larger particles with sizes comparable to the depth of the separation channel cannot reach the ceiling of the main channel, because of the hydrodynamic restriction effect in the widening region, as in the case of the PFF scheme (Figure 1c). Therefore, these large particles frequently enter the separation channel. Meanwhile, small particles can reach the ceiling of the main channel, and they are likely to flow along the main channel and do not often enter the main channel (Figure 1d). Consequently, the difference in the lateral positions (x position) of the particles is enhanced as the particles flow downstream, and these particles are separated based on size. In the following experiments, we examined if this concept explains the mechanism of particle separation using the presented microfluidic systems. 

### 2.2. Fabrication and Design of Microfluidic Devices

Polydimethylsiloxane (PDMS)-glass microfluidic devices were fabricated using standard soft lithography and replica molding techniques [32]. Briefly, we first prepared SU-8 molds on Si wafers. The SU-8 spin-coating and ultraviolet light irradiation processes were repeated twice to obtain dual-depth structures. A PDMS prepolymer (Silpot 184, Dow Corning Toray, Tokyo, Japan) was poured onto the prepared mold, and subsequently cured at 85 °C for 30 min. After completing the crosslinking reaction of PDMS, the PDMS plate with channel structures was peeled off from the mold; subsequently, it was bonded against a flat glass slide (S1112, Matsunami Glass, Tokyo, Japan) after O_2_ plasma-based surface activation. Finally, inlet/outlet silicone tubes were attached and subsequently glued to form the inlet/outlet ports.

**Figure 2:**
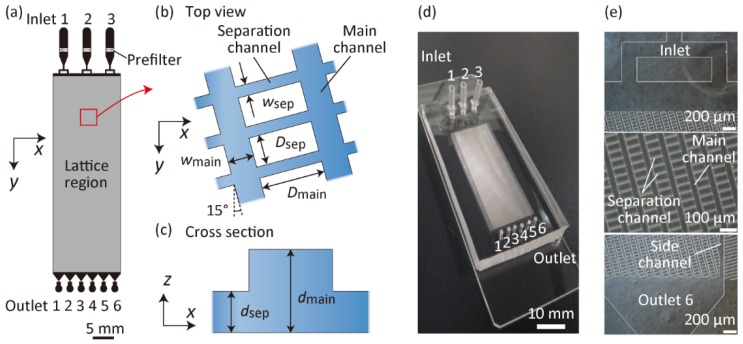
Microchannel design and photographs of the microfluidic devices. (**a**) Top view of Microdevices A–C, (**b**) enlarged image of the lattice region, and **(c**) cross-sectional view of the lattice region. *w*_main_: width of the main channel; *w*_sep_: width of the separation channel; *d*_main_: depth of the main channel; *d*_sep_: depth of the separation channel; *D*_main_: interchannel distance of the neighboring main channels; *D*_sep_: interchannel distance of the neighboring separation channels. (**d**) Photograph of the prepared Microdevice A and (**e**) microscopic images showing the inlet, lattice, and outlet regions of Microdevice A.

We prepared six types of microfluidic devices (Microdevices A-F) with different microchannel geometries, as shown in Figure 2 and Table 1. Each inlet channel was branched into two channels to uniformly introduce fluid samples into the lattice region. Prefilter structures were placed in the inlet channels to avoid the introduction of large particulates into the lattice region. In the lattice region, two types of microchannels crossed perpendicularly. The deeper main channels (width of *w*_main_, depth of *d*_main_) were slanted against the flow direction (*y*-direction) with a slant angle of 15°, and they were periodically placed with the interchannel distance of *D*_main_. The separation channels (width of *w*_sep_, depth of *d*_sep_) were shallower and narrower than the main channels, which were also placed at the interchannel distance of *D*_sep_. Microdevices A–C were prepared to examine the effects of the main channel depth. Microdevices D–F were used to tune the critical size of the particle separation of Microdevice A; these channels exhibited a perfectly similar relationship, but with different sizes. 

### 2.3. Particle Separation Experiments

We employed fluorescent/nonfluorescent standard polystyrene particles with different diameters. Microparticles with the average diameters of 2.1 μm (B0200; blue fluorescent), 3.0 μm (R0300; red fluorescent), 3.1 μm (G0300; green fluorescent), 4.8 μm (G0500; green fluorescent), 6.0 μm (4206A; nonfluorescent), 9.9 μm (G1000; green fluorescent), and 15 μm (4215A; nonfluorescent) were obtained from Thermo Fisher Scientific, MA, USA. These particles were suspended in an aqueous solution of 18% sucrose and 0.5% tween 20 at concentrations of 5 × 10^6^–3 × 10^7^ particles per milliliter. This solution prevents the precipitation of particles. The particle suspension was pumped from Inlet 2, whereas the same solution without particles (the “buffer”) was introduced from Inlets 1 and 3 using syringe pumps (KDS200, KD Scientific, MA, USA). The behaviors of the particles were observed using a fluorescence microscope (IX71, Olympus, Tokyo, Japan) equipped with a charge-coupled device camera (DP80, Olympus, Tokyo, Japan). The absolute numbers of particles, separated and recovered from each outlet, were evaluated by measuring the output volumes and analyzing the particle concentrations using a hemocytometer. The recovery ratio of the particles was defined as the number of particles recovered from an outlet divided by the total particle number recovered from all six outlets. On average, ~200 particles were counted for each condition, and experiments were repeated at least thrice using individual microdevices. To investigate the flow behaviors of microparticles in the *x*-*y* planes at different depths (different *z* positions), a high-speed confocal microscope system (Confocal Scanning Micro PIV System, Seika Corp., Tokyo, Japan) was used. 

## 3. Results and Discussion

### 3.1. Particle Separation Using Microdevice A

In the presented lattice-shaped microchannel networks, the flow rate distribution along the streamline (*y*-axis) is not necessarily uniform because of the asymmetrically placed channels with different depths. The values of the output volumes are key for evaluating the separation efficiency of the target particles. Hence, we first measured the volumetric flow rates distributed to each outlet using Microdevice A, by introducing distilled water from the inlets and measuring the output volumes by weighing. The result is shown in Supplementary Figure S1. As expected, the flow rates through the six outlets were not uniform and a distribution was shown; the flow rate to Outlet 1 was only ~10% of the input flow, whereas that to Outlet 6 was higher (~28%). This non-uniformity is attributable to the relatively wide and deep main channels slanted to the right direction (direction to Outlet 6). For the following particle separation experiments, the absolute number of particles recovered from each outlet was evaluated by multiplying the particle concentration and output volume. 

**Figure 3:**
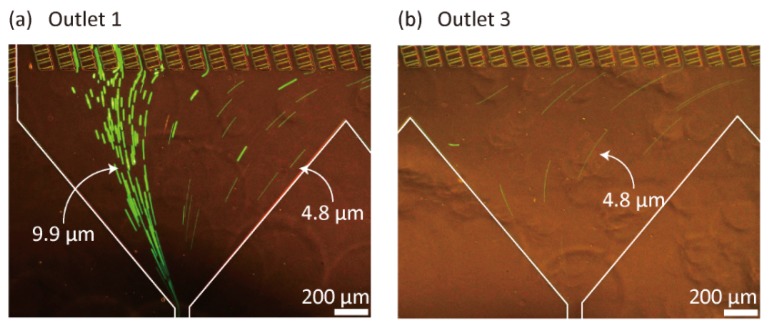
Behaviors of the fluorescent standard particles (4.8- and 9.9-µm green particles) flowing near (**a**) Outlet 1 and (**b**) Outlet 3 of Microdevice A.

Next, we observed the separation behaviors of fluorescent standard microparticles as a model. Figure 3 shows 4.8- and 9.9-µm green particles flowing through Outlets 1 and 3 of Microdevice A, when the input flow rates from Inlets 1, 2, and 3, denoted as *Q*_1_, *Q*_2_, and *Q*_3_, respectively, were 20, 20, and 80 µL/min, respectively. Most of the large 9.9-µm particles flowed through Outlet 1, whereas small 4.8-µm particles were primarily distributed to Outlets 1, 2, and 3. This result clearly indicates that the presented dual-depth lattice-channel network can function as a size-selective sieving matrix for micrometer-sized particles. This separation result is similar to that reported in our previous study using uniform-depth lattice channels [31] or in the DLD scheme [7,8,9], in that the differences in the lateral positions (*x* positions) of particles are enlarged as the particles flow through the lattice region in the *y* direction. However, interestingly, the particle behaviors differ completely from those observed in our previous study because larger particles flow into the separation channels more frequently than the smaller particles, thus resulting in a large degree of lateral displacement distance for the larger particles. 

**Figure 4:**
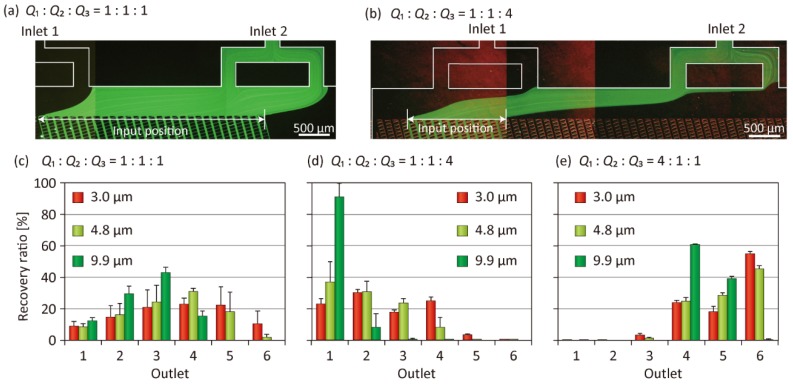
(**a**,**b**) Fluorescence micrographs exhibiting the behaviors of green fluorescent particles flowing into the lattice region of Microdevice A, when the ratios of the input flow rates were changed as indicated. (**c**–**e**) Recovery ratios of three types of particles when the ratio of the input flow rates was changed. The total flow rate *Q*_total_ was constant at 120 µL/min for these experiments. Each set of data represents the mean ± standard deviation (SD) from three individual experiments.

Several key operation parameters may affect the separation performances of the particles using the presented concept. First, the ratio of the input flow rates was naturally regarded as a critical factor. We therefore investigated the effect of the input flow-rate ratio, while maintaining the total flow rate *Q*_total_ (= *Q*_1_ + *Q*_2_ + *Q*_3_) at 120 µL/min. The result is shown in Figure 4. When the ratio of the input flow rates, *Q*_1_, *Q*_2_, and *Q*_3_ was 1:1:1, the input position of particles was relatively broad (~3.8 mm; Figure 4a). The three types of different-sized particles introduced exhibited similar behaviors and were not clearly separated, even though the 9.9-µm particles were not recovered from Outlets 5 and 6 (Figure 4c). Meanwhile, when the relative ratio of the particle suspension (*Q*_2_) was decreased, i.e., when the ratio was changed to 1:1:4 or 4:1:1, the input position was narrowed to ~1.6 mm (Figure 4b). Especially in the 1:1:4 condition, where the particles were introduced from the left-side region of the lattice, a good separation was obtained (Figure 3 and Figure 4d). More than 90% of the 9.9-µm particles were recovered from Outlet 1, whereas 3.0- and 4.8-µm particles were dispersed and primarily recovered from Outlets 1 to 4. Although the separation efficiency was not sufficiently high in this condition, we clarified that a pinching effect of the particle input position had occurred. When the ratio was changed to 4:1:1, that is, the relative value of *Q*_1_ was increased, the introduction position was shifted to the right side of the lattice. In this condition, most of the particles were recovered from Outlets 4 to 6, regardless of the particle size, and particle separation was not achieved (Figure 4e). This result clarified that the input position was a highly critical factor dominating the particle separation performances. A higher separation precision may be expected when the relative value of *Q*_2_ is further lowered; however, we performed particle separation experiments at the input flow rate ratio of 1:1:4 in the following experiments, to ensure a relatively high throughput of particle separation.

In most microfluidic schemes for particle separation using laminar flow systems, the particle separation performances are not significantly affected by the absolute value of the flow rate, provided that the particle inertia is negligible [33]. To examine if the particle separation performance of the presented method is affected by the absolute value of the flow rate, we performed particle sorting experiments under different flow-rate conditions using Microdevice A. The total flow rate *Q*_total_ was changed at 60 µL/min or 1200 µL/min, whereas the flow-rate ratio remained at 1:1:4. The result is shown in Supplementary Figure S2. The ratio of the 9.9-µm particles recovered from Outlet 1 slightly decreased when the total flow rate was increased to 1200 µL/min; the particle separation behaviors did not change significantly compared with those at 60 and 120 µL/min (Figure 4d). This result clearly indicated that the particle inertia did not dominate the separation mechanism of the presented method. Because a relatively high separation throughput was demonstrated, we expect that a high-throughput processing would be possible using the presented concept by further optimizing the operating conditions and/or microchannel geometries, including microchannel parallelization [34].

Additionally, we attempted to observe the flowing behaviors of mammalian cells to validate the applicability of the presented method for cell sorting/manipulation applications. We introduced a suspension of NIH-3T3 cells in phosphate buffered saline (PBS), whose nuclei were stained blue using Hoechst 33342 dye, from Inlet 2, and PBS without cells from Inlets 1 and 3. The input flow rates *Q*_1_, *Q*_2_, and *Q*_3_ were 20, 20, and 80 µL/min, respectively. The results indicate that these cells, with an average diameter of ~12 µm, were mostly recovered from Outlet 1 (Supplementary Figure S3), as in the case of the 9.9-µm particles. Although we did not perform size-based fractionation of cells with specific sizes, the cell concentration was increased approximately twice after recovery from Outlet 1. This result demonstrates applicability to cell separation in addition to cell concentration and carrier medium exchange using the presented microfluidic device. 

### 3.2. Observation of 3D Particle Behavior Using High-Speed Confocal Microscopy

**Figure 5:**
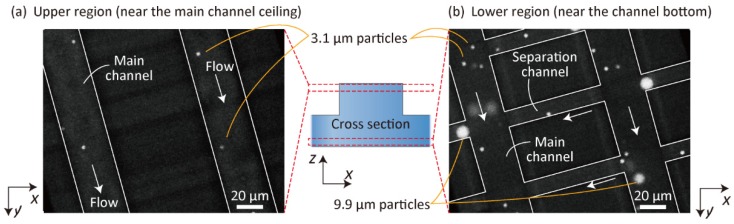
High-speed confocal microscopic images showing the flow behaviors of 3.1- and 9.9-µm particles (**a**) in the upper region (near the main-channel ceiling) and (**b**) in the lower region (near the channel bottom). Small circular dots indicate 3.1-µm particles, whereas the large circular objects indicate 9.9-µm particles. The microchannel is visualized by the white lines.

To elucidate the separation mechanisms in detail and to strengthen our theory as depicted in Figure 1, we observed the behaviors of green fluorescent 3.1- and 9.9-µm particles in the lattice region using high-speed confocal microscopy. A buffer solution and the particle suspension were introduced into Microdevice A at *Q*_1_, *Q*_2_, and *Q*_3_ of 2.0, 2.0, and 8.0 µL/min, respectively. Particles flowing near the ceiling of the main channel and those near the bottom surface were individually observed. The flows of the particles are shown in Figure 5 and Supplementary Video S1. Near the ceiling of the microchannel, we primarily observed 3.1-µm particles flowing along the main channel, but the number of large 9.9-µm particles was extremely small. It was assumed that the large particles could not reach the ceiling of the microchannel when they were flowing from the shallower separation channel into the deeper main channel, because of the hydrodynamic constriction effects for the large particles, as in the case of PFF [10]. By contrast, 9.9- and 3.1-µm particles, flowing near the bottom surface, frequently entered the separation channels (Figure 5b). Furthermore, we observed that 3.1-µm particles flowed in the upper and/or lower direction (+*z* or -*z* direction) more frequently than the 9.9-µm particles. Once the small particles reached the ceiling of the main channel, they flowed along the main channel. These observations clarified that the separation mechanism of this scheme utilized 3D flows in the planar lattice-shaped microchannel networks, as explained in Figure 1. 

### 3.3. Effect of the Main Channel Depth on Particle Separation

**Figure 6:**
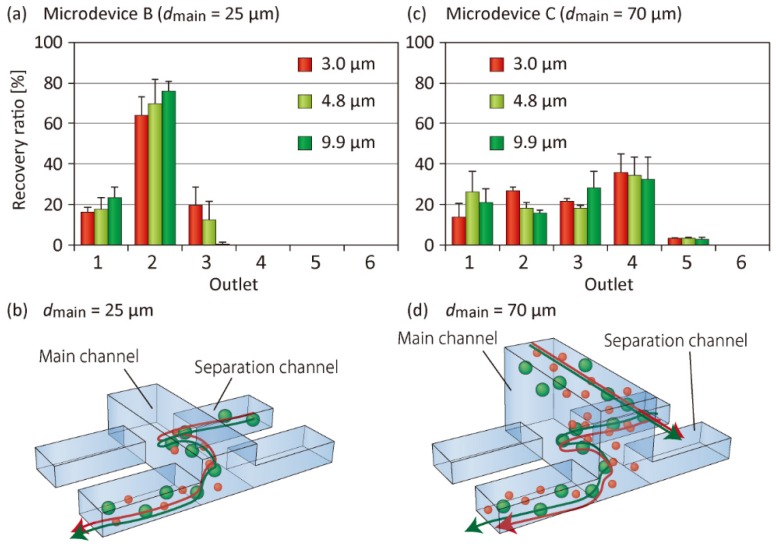
Recovery ratios of three types of microparticles and schematic images showing the particle behaviors in the dual-depth lattice channels. (**a**,**b**) Microdevice B with the main channel depth of 25 µm and (**c**,**d**) Microdevice C with the main channel depth of 70 µm. In (**a**,**c**), each set of data represents the mean ± SD from three individual experiments.

In addition to the operation parameters, various geometric parameters of the microchannels affect the separation behaviors of the particles. Among various factors, we investigated the effect of the main channel depth *d*_main_ on particle separation performances. In addition to Microdevice A with *d*_main_ of 40 µm, we fabricated two microdevices, Microdevices B and C, with *d*_main_ values of 25 and 70 µm, respectively (Table 1). The results of particle separation are shown in Figure 6. In Microdevice B, the three types of particles introduced were primarily recovered from Outlet 2, and significant differences were not observed between these particles (Figure 6a). This result suggested that the particle migration in the upper (*z*) direction was suppressed, because the particles, once introduced from the separation channel into the main channel, could easily be reintroduced into the separation channel (Figure 6b). By contrast, when Microdevice C was used, the particles were almost randomly distributed to Outlets 1–4 (Figure 6c). It was likely that the particles, once they had reached near the ceiling of the main channel, flowed along the main channel and never flowed near the channel bottom, and were recovered from Outlets 3–4. Meanwhile, particles flowing near the bottom surface would be frequently introduced into the separation channels, regardless of the particle size (Figure 6d). Consequently, we confirmed that Microdevice A with *d*_main_ of 40 µm was the optimal device among the three types of devices for separating microparticles with this size range. 

### 3.4. Tuning of Separation Size

**Figure 7:**
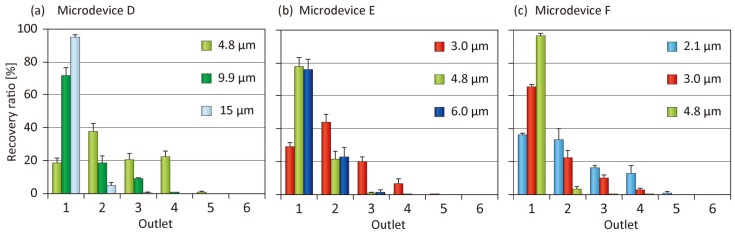
Separation results of model particles with different similitude ratios; (**a**) similitude ratio was 1.5 (microdevice D), (**b**) similitude ratio was 0.66 (microdevice E), and (**c**) similitude ratio was 0.5 (microdevice F). The *Q*_total_ and Q_1_:Q_2_:Q_3_ were 120 µL/min and 1:1:4, respectively. Each dataset presents the mean ± SD from three individual demonstrations.

The ability to tune the separation size is significantly important for a microfluidic particle separation scheme to widen its application range. It may be possible to alter one or more geometrical parameters of Microdevice A to tune the separation size; however, we prepared three additional types of microfluidic devices (Microdevices D, E, and F), all of which exhibited a perfectly similar relationship with Microdevice A. The relative sizes of Microdevices D, E, and F to Microdevice A were 1.5, 0.67, and 0.5, respectively. We performed particle separation experiments with the input flow rates *Q*_1_, *Q*_2_, and *Q*_3_ of 20, 20, and 80 µL/min, respectively, for all the devices. To examine the controllability of the separation size, we used several types of fluorescent and/or nonfluorescent model particles with a diameter from 2.1 to 15 µm. The results are shown in Figure 7. For each microdevice, the largest particles were primarily recovered from Outlet 1, whereas the smallest particles were distributed to Outlets 1 to 4, as in the case of Microdevice A. For example, 4.8-µm particles were distributed to Outlets 1–4 of Microdevice D, but were mainly recovered from Outlet 1 of Microdevice F. These results clearly suggest that the usage of different-sized microfluidic devices in a similar relationship is a reasonable strategy to effectively tune the separation behaviors of the particles. 

### 3.5. Control of the Vertical Position of Particles

**Figure 8:**
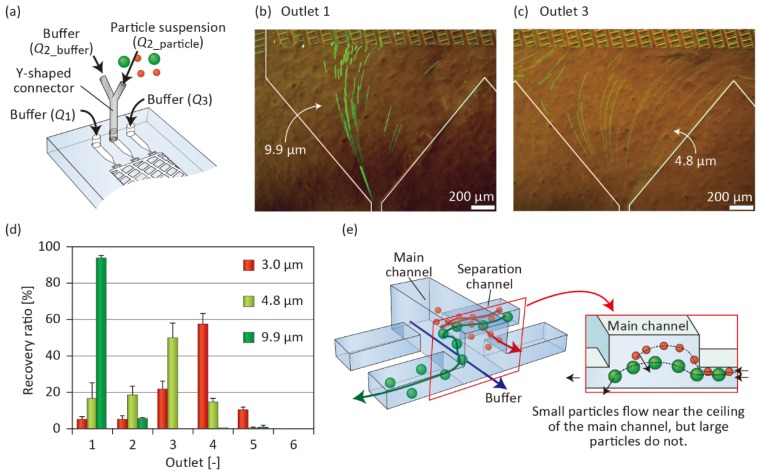
(**a**) Schematic image showing Microdevice A with an attached Y-shaped connector. (**b**,**c**) 4.8- and 9.9-µm particles flowing near Outlets 1 and 3. (**d**) Result of particle separation when *Q*_1_, *Q*_2_particles_, *Q*_2_buffer_, and *Q*_3_ were 20, 2, 18, and 80 µL/min, respectively. Each set of data represents the mean ± SD from three individual experiments. (**e**) Schematic image showing the particle behaviors in the lattice channel, when the input position of the particles was restricted to the upper region.

From the results on particle separation and observations, we assumed that the difference in particle position in the *z* direction might be the primary reason for the insufficient separation efficiency. This could be especially severe for the smaller particles introduced; large particles were primarily collected from Outlet 1, but smaller particles were distributed to Outlets 1–4. To improve the precision of particle separation, we employed a Y-shaped connector that was inserted into Inlet 2 of Microdevice A (Figure 8a). The particle suspension was introduced from one of the two branch inlets, whose position was close to the outlets (+*y* direction), whereas the buffer without particles was introduced from another inlet, which was close to the inlets (−*y* direction). Using such a setup, almost all the particles were pushed against the ceiling of the microchannel in the lattice region. The flow rates *Q*_1_, *Q*_2_particles_, *Q*_2_buffer_, and *Q*_3_ were 20, 2, 18, and 80 µL/min, respectively. 

Figure 8b,c show the 4.8- and 9.9-µm particles flowing near Outlets 1 and 3, respectively. Compared with the separation results without using the Y-shaped connector (Figure 3), the number of 4.8-µm particles flowing through Outlet 1 decreased significantly. The separation results of the three types of particles are shown in Figure 8d. The introduced 3.0-, 4.8-, and 9.9-µm particles were mainly recovered from Outlets 1, 3, and 4, respectively. The 3.0- and 4.8-µm particles were separated, indicating that the separation resolution had improved. This result indicated that the small particles were likely to selectively flow near the ceiling of the main channel, whereas the large particles, flowing near the bottom, frequently entered the separation channels (Figure 8e). The separation resolution of the 3.0- and 4.8-µm particles was not high in these experiments, possibly because these particles are relatively small compared to the critical separation size of the presented microfluidic device. A possible strategy to improve the separation resolution of these particles is to sequentially employ several types of microfluidic devices with different separation sizes. From these results, we confirmed that the introduction of particles from the limited area in the *x*-*z* plane of the lattice region was a highly effective strategy to improve the separation precision of dual-depth, asymmetric microchannel systems. Although the throughput of separated particles decreased compared with the result shown in Figure 3, the presented approach could offer useful insights into microfluidic particle/cell sorting using 3D flow profiles in stereoscopic microchannel structures. 

## 4. Conclusions

We have successfully demonstrated a new concept of a microfluidic particle separation scheme using dual-depth, asymmetric microfluidic lattice structures. By precisely observing the separation behaviors of model particles, we revealed that the 3D laminar flow profile was utilized for the size-selective differences in particle behaviors. Additionally, the control of the input position in both *x* and *z* directions was significantly effective in improving the separation performances. The lattice configuration was advantageous because it was robust against microchannel clogging, as bypass flows were generated even when microchannels were clogged at several points. With the recent progress in 3D fabrication techniques, including 3D printers, microfluidic channels with complex 3D structures have recently been gaining increasing attention. The results obtained in this study would provide useful insights into the utilization of 3D flows in microchannels for the separation and manipulation of various types of micrometer-sized particles. 

## Figures and Tables

**Table 1:** Parameters of six types of fabricated microfluidic devices. The parameters correspond to those shown in Figure 2. The size ratio indicates the relative size ratios for Microdevices A, D, E, and F.

Microdevice	*w*_main_ (μm)	*w*_sep_ (μm)	*d*_main_ (μm)	*d*_sep_ (μm)	*D*_main_ (μm)	*D*_sep_ (μm)	Lattice Size (mm × mm)	Size Ratio
A	35	15	40	15	93	25	32 × 12	1
B	35	15	25	15	93	25	32 × 12	-
C	35	15	70	15	93	25	32 × 12	-
D	52	23	60	23	139	38	48 × 18	1.5
E	23	10	27	10	62	17	21.3 × 8	0.67
F	17	7	20	7	46	13	16 × 6	0.5

## Supplementary Materials

The following are available online at https://www.mdpi.com/2072-666X/10/6/425/s1: Figure S1: The output volumes from the six outlets of Microdevice A; Figure S2: Results of particle separation using Microdevice A when the total flow rate was changed; Figure S3: Fluorescence micrograph showing the behaviors of mammalian cells in Microdevice A; Video S1: High-speed confocal microscopic movie showing the flowing behaviors of 3.1- and 9.9-µm particles in Microdevice A, which correspond to those shown in Figure 5.
