# Supplementary material for: Hydrodynamic Microparticle Separation Mechanism Using Three-Dimensional Flow Profiles in Dual-Depth and Asymmetric Lattice-Shaped Microchannel Networks
Source: Micromachines (Basel). 2019 Jun 25;10(6):425. doi: 10.3390/mi10060425 (PMC6632020; doi:10.3390/mi10060425)
Supplement: Supplementary file 1 [file micromachines-10-00425-s001.zip › micromachines-533034-SI/Supplementary Information.docx]

Supplementary Materials: Hydrodynamic Microparticle Separation Mechanism Using Three-dimensional Flow Profiles in Dual-depth and Asymmetric Lattice-shaped Microchannel Networks

Takuma Yanai ^†^, Takatomo Ouchi ^†^, Masumi Yamada * and Minoru Seki

Department of Applied Chemistry and Biotechnology, Graduate School of Engineering, Chiba University, 1-33 Yayoi-cho, Inage-ku, Chiba 263-8522, Japan; lyushi.15m@gmail.com (T.Y.); tktm888@gmail.com (T.O.); mseki@faculty.chiba-u.jp (M.S.)

***** Correspondence: m-yamada@faculty.chiba-u.jp; Tel.: +81-43-290-3398

^†^ These authors equally contributed to this work


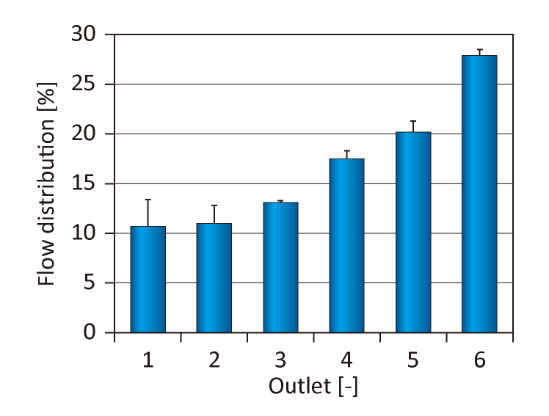


**Figure S1.** The output volumes from the 6 outlets of Microdevice A, when the input flow rates *Q*_1_, *Q*_2_, and *Q*_3_ were 20, 20, and 80 µL/min, respectively. Each data represents the mean ± SD from 3 individual experiments.


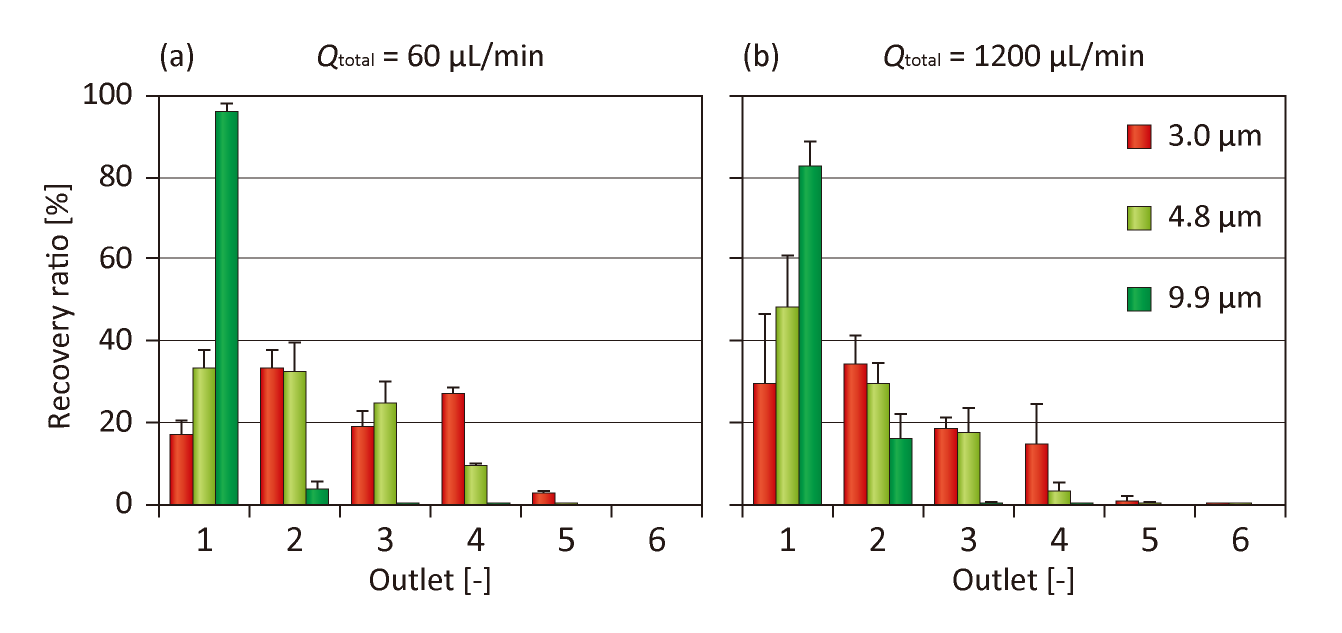


**Figure S2.** Results of particle separation using Microdevices A when the total flow rate was changed at (**a**) 60 µL/min and (**b**) 1200 µL/min, while keeping the flow-rate ratio constantly at 1:1:4. Each data represents the mean ± SD from 3 individual experiments.


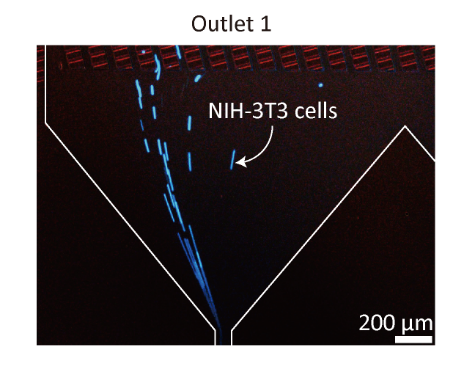


**Figure S3.** Fluorescence micrograph showing the behaviors of mammalian cells in Microdevice A, when the input flow rates *Q*_1_, *Q*_2_, and *Q*_3_ were 20, 20, and 80 µL/min, respectively. NIH-3T3 cells (mouse fibroblasts) were suspended in phosphate buffered saline at a concentration of 1 × 10^6^ cells/mL, and the cell suspension was introduced from Inlet 2. The cell nucleus was stained blue with Hoechst 33342 dye. Almost all the cells were recovered from Outlet 1.
